# Enhancing museum collection images with fuzzy set guided convolutional neural network: A novel approach leveraging fuzzy set theory

**DOI:** 10.1371/journal.pone.0336426

**Published:** 2025-11-17

**Authors:** Manqi Li, Lili Ren

**Affiliations:** 1 School of Fine Arts and Design, Heze University, Heze, Shandong, China; 2 Solux School of Architecture and Design, University of South China, Hengyang, Hunan, China; Dayananda Sagar University, INDIA

## Abstract

Museum collection images are invaluable for preserving cultural heritage and studying history. However, these images often lack quality and clarity. This study introduces a novel museum collection image enhancement technique based on fuzzy set theory. The proposed approach comprehensively addresses the complexity and uniqueness of museum collection images to improve their quality significantly. The methodology steps start with preprocessing and graying out the images, eliminating noise through segmentation and smoothing processes. A convolutional neural network (CNN) is utilized to extract image features and apply adaptive histogram equalization for enhancement. A distinctive aspect of our method is transforming image grayscale levels into fuzzy sets. We analyze the similarity between the fuzzy sets before and after enhancement using the cosine similarity algorithm, allowing us to reconstruct the processed images with targeted similarity. Testing our approach on 100 museum collection images, we found that the average contrast of the collection images improved significantly. Specifically, the average contrast for our fuzzy set-based enhancement was 0.91, compared to 0.81 and 0.79 for histogram equalization and wavelet transform methods, respectively. Our research showcases a museum collection image enhancement technology based on fuzzy set theory that effectively enhances image fidelity and clarity, improving the overall quality of museum collection images. Our work underscores the importance of ongoing research in this area to unlock the full potential of museum collection image enhancement technology.

## Introduction

Museums protect the artifacts from the past and serve as windows into the past, as the strongholds of human cultural legacy. These archives act as educational lighthouses, teaching current and future generations about the rich fabric of our cultural and historical heritage. Museums have been increasingly adopting digital technology in their admirable efforts to preserve this invaluable legacy and make it easier to study. With the help of this technological transformation, museums can now store, preserve, and handle a multitude of data related to their cultural treasures, providing a virtual shield of protection for these historical artifacts [1]. Museum collections are an important carrier of cultural heritage, and through the display of collections, museums can inherit historical culture and play a role in cultural education [2]. Museum collections are also important resources for studying history and culture. By analyzing the characteristics of collections, one can gain a deeper understanding of history and culture. The photos and images of museum collections are important materials for recording history. Due to the relatively old age of museum collections, they are subject to natural wear and tear. Under the interference of factors such as light, dust, and shooting equipment, the quality of museum collections’ images varies. The aging of museum collection images and human destruction are also the main reasons that affect the quality of collection images. Historical and cultural studies and the transmission of historical literature are adversely impacted by the poor quality of visual resources in museum collections. The necessity of improving and returning these photos to their previous splendor is highlighted by the deterioration in image quality and the continued effects of degradation caused by humans.

Traditional CNN mainly relies on deterministic convolution kernels and fixed weights to extract features, which makes it challenging to adapt to the ambiguity and uncertainty in image data, especially in the case of illumination changes, noise interference, or low contrast. Fuzzy set theory can effectively model the uncertainty in images and normalize the pixel grayscale values through fuzzy membership functions to make image features more robust. In addition, fuzzy sets can be combined with CNN to provide more flexible feature expressions, improve the model’s adaptability to complex visual data, and thus optimize the performance of image enhancement and classification tasks. Picture enhancement technology offers a potentially effective way to overcome these complex problems. This technology revitalizes the photographs in museum collections by utilizing various processes, such as denoising, restoration, and other image-processing approaches. This renewal is crucial in ensuring the pictures stay loyal to the authentic nature of the cultural objects they portray, not just cosmetic ones.

The field of image enhancement technology is always evolving, and many processing techniques, like denoising and mending, can enhance the quality of images. Improved photos are essential resources for preserving and sharing knowledge about cultural heritage. These pictures are more than just documentation tools; they also serve as information conduits, bringing the spirit of cultural artifacts to a broader audience. These artifacts’ aesthetic appeal increases their instructional worth, which makes them perfect for cultural outreach and education. Take a look at the work done by Sugiura et al. [3] to see the potential of image enhancement. They applied augmented reality technology to medical specimen museum visits, using cameras to obtain medical specimen images and utilizing augmented reality technology to improve the quality of medical specimen images. Through dissection and slicing, the specimens in these museums allow students to appreciate the complex relationships between organs and structures in greater detail than in textbooks. Zhao [4,5] used virtual reality technology to construct a personalized healthcare museum, which enhanced image quality and enhanced the cultural dissemination role of healthcare museums by processing exhibition images in the museum. Hackenbroich et al. [6,7] described the Vindolanda Museum’s development of the “Mining Memories - Making Connections” digital exhibition, which showcases their responses through creative writing, audio recordings, and short film clips, with 3D models of selected artifacts embedded for enhancement. Budge and Burness [8,9] pointed out that museums focus on engaging the public in collections, exhibitions, and projects, interacting with mass media through displaying collection images, and using photography to exchange and share museum collection experiences. Image enhancement processing of museum collection images can improve the collection images’ clarity and preserve the collection’s authenticity as much as possible. However, too many uncertain factors affect the image quality of museum collections, and there is a lack of consideration for the complexity of collection images.

The images of museum collections are affected by factors such as lighting and shooting equipment, and the display effect of museum collections is poor. Improving the restoration of museum images is very important. To realize the digital watermarking technology for the copyright protection of museum collections, Wang et al. [10] used SIFT technology to restore the geometric transformation distortion (rotation, scaling, and translation) of museum digital image exhibits, and achieved good results, laying a foundation for the application research of copyright protection based on digital watermarking technology. The importance of digital technology in the museum industry has been brought to light by the difficulties presented by the COVID-19 epidemic. Museums have taken advantage of this quickly changing environment to better engage with their audiences by utilizing digital displays and picture technology. In particular, during the pandemic, Taormina and Baraldi emphasized the critical role that digital technology had in improving the quality of image presentations and, as a result, the communication efficacy of museum information [11].

When the ambiguity of an image is high, it is necessary to consider the complex characteristics of the image. Villaespesa and Murphy [12] applied computer vision to museum collections, showcasing traditional museum collections in images. Through image denoising and other processing, the image quality of museum collections was improved, providing a new way to develop museums. By visualizing museum collections and enhancing their image quality through image technology, the dissemination effect of museum collections can be improved. However, insufficient consideration is given to the complexity of collection images and the lack of respect for specific image lighting and other factors. Aiming at the defects that fuzzy enhancement cannot improve the contrast of images and histogram equalization enhancement has the defects of over-enhancement and loss of image detail information, the image enhancement algorithm based on intuitionistic fuzzy sets and histogram equalization is realized by synthesizing the intuitionistic fuzzy membership and non-membership of pixels. It can significantly enhance the detailed information of the image but cannot improve the image contrast [13].

Factors such as lighting severely affect the clarity and restoration of museum collections’ images. After denoising and feature extraction, the fuzzy set similarity algorithm is used to analyze and process the collection of images to improve their quality.

A review of related research is shown in Table 1.

**Table 1:** Review of related research.

References	Datasets	Methods	Analysis parameters	Main findings
Sugiura et al. (2019) [3]	Medical specimen museum images	Augmented reality technology	Brightness, clarity	Improving the image quality of medical specimens through AR technology
Zhao (2023) [4]	Personalized medicine museum images	Virtual reality + deep learning	Color enhancement, feature extraction	Improving the display effect of museum images through VR
Hackenbroich et al. (2023) [6]	Vindolanda museum images	3D modeling and digital exhibition	Texture, structural similarity	Improving the interactivity and display quality of digital exhibitions
Wang et al. (2010) [10]	Digital museum exhibit images	SIFT digital watermarking	Geometry transformation correction	Improving the copyright protection ability of museum images through digital watermarking
Villaespesa & Murphy (2021) [12]	Computer vision museum images	Computer vision technology	Detail enhancement, denoising	Improving the quality of museum images through computer vision
This study	100 museum collection images	Fuzzy set + CNN	Brightness, contrast, similarity	Using a fuzzy set algorithm to optimize image quality and improve image clarity

Innovatively advancing museum collection image enhancement, our research introduces a pioneering approach rooted in fuzzy set theory, addressing the intricate challenges posed by the complexity and uniqueness of these historical artifacts. Leveraging a combination of preprocessing, noise elimination, and feature extraction powered by a convolutional neural network, our methodology transforms grayscale levels into fuzzy sets, allowing for a nuanced analysis of image similarity before and after enhancement using the cosine similarity algorithm. This distinctive technique facilitates targeted enhancement of dissimilar pixels, substantially improving the overall quality and clarity of museum collection images. Our comparative performance analysis underscores the superiority of our fuzzy set-based enhancement, particularly in achieving a remarkable average contrast improvement compared to traditional methods such as histogram equalization and wavelet transform. As a noteworthy contribution to the field, our work not only showcases the efficacy of our strategy but also emphasizes the importance of further research in this domain, pointing towards new horizons for digital preservation and the inheritance of cultural knowledge embedded in museum artifacts.

## Materials and methods

This research’s methodology is centered on a new fuzzy set theory approach that stands out for its capacity to adjust to the intricacy and individuality of photographs from museum collections. Comparing this process to other ways, there are some significant benefits. First, we may more accurately depict and portray the innate ambiguity and uncertainty seen in photos from museum collections by utilizing fuzzy set theory. This is especially important because many variables affect how unclear these photos are, such as illumination and the deterioration of the image over time. When considering improvement techniques, the fuzzy set-based approach offers greater flexibility and context awareness than traditional techniques such as wavelet transformations or histogram equalization [14].

It is remarkable how well this technique fits the particular goals of our study. Our main objective is to improve the clarity and quality of photos from museum collections to aid in preserving cultural heritage. The fuzzy set theory facilitates the examination and conversion of pixel gray values into matching fuzzy sets, which maintain the distinctiveness of these pictures while collecting minute details [15]. We accomplish targeted picture improvement by identifying dissimilar pixels using the cosine similarity technique.

### Image preprocessing

Museums are gatherings of historical and cultural heritage, spreading heritage culture through displaying collections [16]. Museum collections of cultural relics are carriers of cultural heritage. After being affected by lighting conditions, camera settings, damage, or degradation, museum collection images often exhibit low quality, low contrast, and much noise.

Denoising and enhancing the image quality of museum collections can help better inherit culture. Traditional image enhancement technology is often based on pixel-level operations, such as histogram equalization, wavelet transform, etc., which enhance the image’s visual effect through local brightness and smooth adjustment of image pixels [17]. Traditional image enhancement techniques cannot process complex information and extract effective features.

To carry out image enhancement analysis on museum collection images, it is necessary to acquire a large number of museum collection image data sets and collect museum collection images on the official website of the National Museum of China. Before performing image enhancement on museum collections, it is necessary to preprocess the images. Image preprocessing minimizes the noise data in the original image as much as possible, which is beneficial for subsequent feature extraction [18].

The images of museum collections are preprocessed, including noise removal, image segmentation, and image smoothing. Noise removal refers to the removal of noise introduced during the image acquisition or transmission process, improving the image’s signal-to-noise ratio and highlighting the image’s detailed features. Image segmentation divides an image into different regions, which is beneficial for feature detection. Image smoothing makes the image pixels smoother and eliminates anomalies, which is helpful for image feature extraction [19].

To process the images of museum collections, first of all, it is necessary to convert color images into grayscale. The original images are composed of red, green, and blue colors. Image graying can avoid band distortion. The formula for grayscale processing is as follows:


Gray(i,j)=0.229×R(i,j)+0.587×G(i,j)+0.114×B(i,j) (1)

After grayscale processing of museum collection images, image segmentation is required. Threshold segmentation is the process of binarizing the pixels in the image and dividing the image into two parts according to a certain threshold. The formula for threshold segmentation is expressed as:


$$K(i,j)=\left\{ \begin{array}{c} \text{2}\text{5}\text{5},\;Gray(i,j)>t\\ \text{0},\;Gray(i,j)\leq t \end{array}\right.\;$$ (2)

In Formula (2), *t* represents the threshold for image segmentation.

The original museum collection images contain a large amount of noise data, and mean filtering can be used to average the pixels in the surrounding area, thereby filtering out most of the noise. The formula for mean filtering is expressed as:


$$P(i,j)=\frac{\text{1}}{{\left(\text{2}n+\text{1}\right)}^{\text{2}}}\sum_{v=j-n}^{\text{1}+n}\sum_{u=i-n}^{i+n}P(u,v)$$ (3)

In Formula (3), the sliding window for mean filtering processing is (2n+1)×(2n+1)(2n+1)×(2n+1).

**Fig 1:**
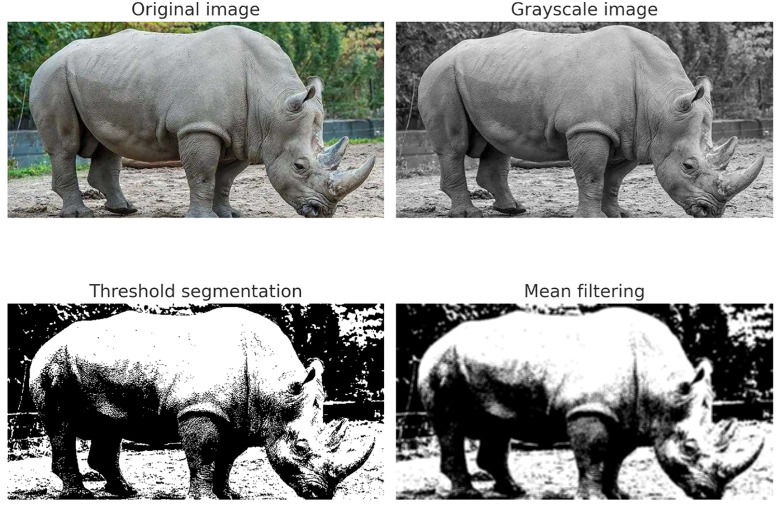
Image preprocessing results of rhinoceros images (original, grayscale, threshold segmentation, and mean filtering). Republished from images.cv, Rhinoceros Image Classification Dataset under a CC BY 4.0 license, with permission from images.cv, original copyright 2020.

The results of preprocessing museum collection images are shown in Fig 1.

In Fig 1, the image preprocessing results of museum collections are described. The original museum collection images are processed with grayscale processing, threshold segmentation, and mean filtering, which can eliminate the noise data in the original image as much as possible, improve the signal-to-noise ratio of the image, and provide favorable assistance for subsequent collection image feature extraction.

### Image feature extraction

Due to factors such as lighting, shooting equipment, and shooting age, the clarity of museum collection images is often not high, which leads to poor performance of museum collections displayed through images. Enhancing the collection images is beneficial for researchers to observe and analyze the collection better and for disseminating heritage culture. Before image enhancement, it is necessary to extract the features of the collection image through feature extraction methods, mainly including texture features, grayscale features, edge features, etc., to improve the analysis and understanding ability of the collection images. Convolutional Neural Networks (CNNs) are deep learning models specifically designed for processing and analyzing visual data, such as images and videos. The methodology of a typical CNN involves several key steps. It starts with input data in the form of two-dimensional grids of pixels, representing images. The heart of the CNN is the convolutional layer, comprising learnable filters that slide over the input image, extracting feature maps that capture specific patterns, like edges and textures. The convolutional neural network can extract image features [20–22]. CNN can perform inference on new, unseen data. Input images are fed through the network, and the output layer’s activations represent the model’s predictions. The combination of convolutional layers, pooling layers, and fully connected layers, along with the ability to learn hierarchical representations, empowers CNNs to excel in computer vision tasks. They automatically learn relevant features from the data, making them powerful tools for image recognition, object detection, and more, with the capability to generalize well to unseen examples.

In this method, CNN extracts images’ hierarchical features (such as edges, textures, etc.) through sliding window operations of multi-layer convolution kernels. Its parameters are optimized through backpropagation, which complements the fuzzy set mechanism rather than directly coupled. The core role of fuzzy set theory is reflected in the feature selection stage: The feature map extracted by CNN is transformed by fuzzy sets, and the pixel grayscale value is mapped to the membership function to construct a fuzzy feature space. In this space, the “uncertainty threshold” is defined as a dynamic benchmark for fuzzy similarity measurement, and the cosine similarity algorithm quantifies the difference between the original image and the enhanced image features. When the fuzzy similarity of the feature area is lower than the preset threshold, the system determines that there is significant information loss or noise interference in the area and then triggers the adaptive enhancement mechanism – instead of adjusting the convolution kernel parameters of CNN, the feature map is nonlinearly weighted through the fuzzy rule base to strengthen the feature response of the low-similarity area. This staged processing strategy not only retains the feature learning ability of CNN but also realizes the precise control of the uncertain regions through fuzzy logic, forming a closed-loop optimization process of “feature extraction-fuzzy evaluation-selective enhancement.”

The steps of image feature extraction by convolutional neural network are as follows. Museum collection images are taken as input. The convolution layer can extract the preliminary feature information in the collection images, and the convolution core can traverse the input collection images by sliding, thus obtaining a two-dimensional convolutional feature map.

The convolutional kernel has the exact dimensions of the input image but on a smaller scale. If the dimension of the input museum collection image is 5×5×15×5×1, where 1 represents a single channel, then the size of the convolutional kernel can be 3×3×13×3×1 When there are n feature extraction convolutional kernels in the convolutional layer, n feature maps are generated, and the step size is the unit of each movement of the convolutional kernel.


$$M=\frac{N-m}{s}+\text{1}$$ (4)

In Formula (4), *N* represents the scale of the input collection image and *M* represents the dimension of the feature map. *m* represents the scale of the convolutional kernel and *s* represents the sliding step size of the convolutional kernel.

The feature map can be represented as:


Y=X×W (5)

In Formula (5), *X* represents the input collection image and *W* represents the convolution of the convolution kernel.

After the image is convolved, many feature maps are generated. Their dimensionality is generally high. To retain important feature information, it is necessary to reduce their dimensionality through pooling layers. The pooling layer sets multiple pooling windows and selects the maximum value within the window as the output, effectively reducing the dimensionality of feature data.

In convolutional neural network training, the weight of the convolutional kernel can be adjusted through error backpropagation to achieve the purpose of accurate feature extraction. The form of error is expressed as:


$$E=\frac{\sum_{j}\;{\left(y_{\text{1}j}-y_{\text{2}j}\right)}^{\text{2}}}{\text{2}}$$ (6)

In Formula (6), $y_{\text{1}j}$ and $y_{\text{2}j}$ represent the actual output and expected output of the $j^{th}$ output neuron, respectively.

Let e be the maximum acceptable error value. When E≤e, it means that the convolutional neural network has highly accurate collection image feature extraction.

The texture features of museum collection images reflect the texture and structural information of the collection. Through the collection’s texture information, the collection type can be analyzed, and targeted image enhancement processing can be carried out.

### Fuzzy set processing

The fuzzy set is a concept in fuzzy theory, which is composed of multiple fuzzy sets and can handle fuzzy relations and uncertainties in logic well [23]. The cosine similarity algorithm can be used to calculate the similarity between two fuzzy sets of images. The formula for the cosine similarity algorithm is:


$$OS\theta =\frac{\left(A.B\right)}{\left\|A\right\|\times \left\|B\right\|}$$ (7)

In Formula (7), *A* and *B* represent two image vectors, respectively. The cosine similarity value is [−1,1], and the larger the value, the more similar it is.

Analyzing the degree of similarity of the original image before and after enhancement judges the effect of improving the quality of the collection image. Enhancing the feature information of the collection image specifically, the similarity function is used to evaluate the impact of image enhancement. If the cosine similarity value is close to 1, it indicates that image enhancement can preserve many data features of the original collection images while improving image quality and visual effects.

The enhancement of museum collection images requires a higher granularity image enhancement method to improve the quality and information of museum collection images. The processing of the fuzzy set can analyze the similarity of the original collection images before and after image enhancement through the similarity function, identify areas with similar features, and improve the quality of museum collection images through targeted enhancement adjustments.

In the CNN structure, the fuzzy membership function is combined with the traditional convolution operation through the adaptive fuzzy convolution layer to enhance the network’s adaptability to uncertain data. Specifically, in the input layer, the pixel values are normalized by the fuzzy membership function, and the input data is converted into the member values of different fuzzy sets better to represent the fuzziness and subtle changes in the image. In the convolution layer, a fuzzy weighting mechanism is introduced to dynamically adjust the weights of the convolution kernel through fuzzy rules so that it pays different attention to features in other areas. In low-contrast areas, the fuzzy convolution kernel can adaptively enhance edge details, while in high-noise areas, the fuzzy filter can suppress irrelevant information, thereby improving the robustness of CNN in complex visual environments.

In addition, in the pooling layer and feature extraction stage, fuzzy logic operations can be used to improve the perception of CNN for multi-scale features. Compared with traditional maximum pooling or mean pooling, fuzzy pooling uses a fuzzy aggregation function to dynamically adjust the response value of the pooling window, thereby reducing information loss and improving feature expression capabilities. In the feature fusion stage, the fuzzy inference system can be used to calculate the fuzzy similarity of high-level features and adjust the learning strategy of the network. The attention mechanism is optimised by calculating the fuzzy similarity of features from different channels, allowing CNN to extract key features more accurately.

### Image reconstruction

Histogram equalization improves the clarity and authenticity of an image by equalizing the distribution of image pixels among the relatively concentrated gray areas in the gray histogram [24].

**Fig 2:**
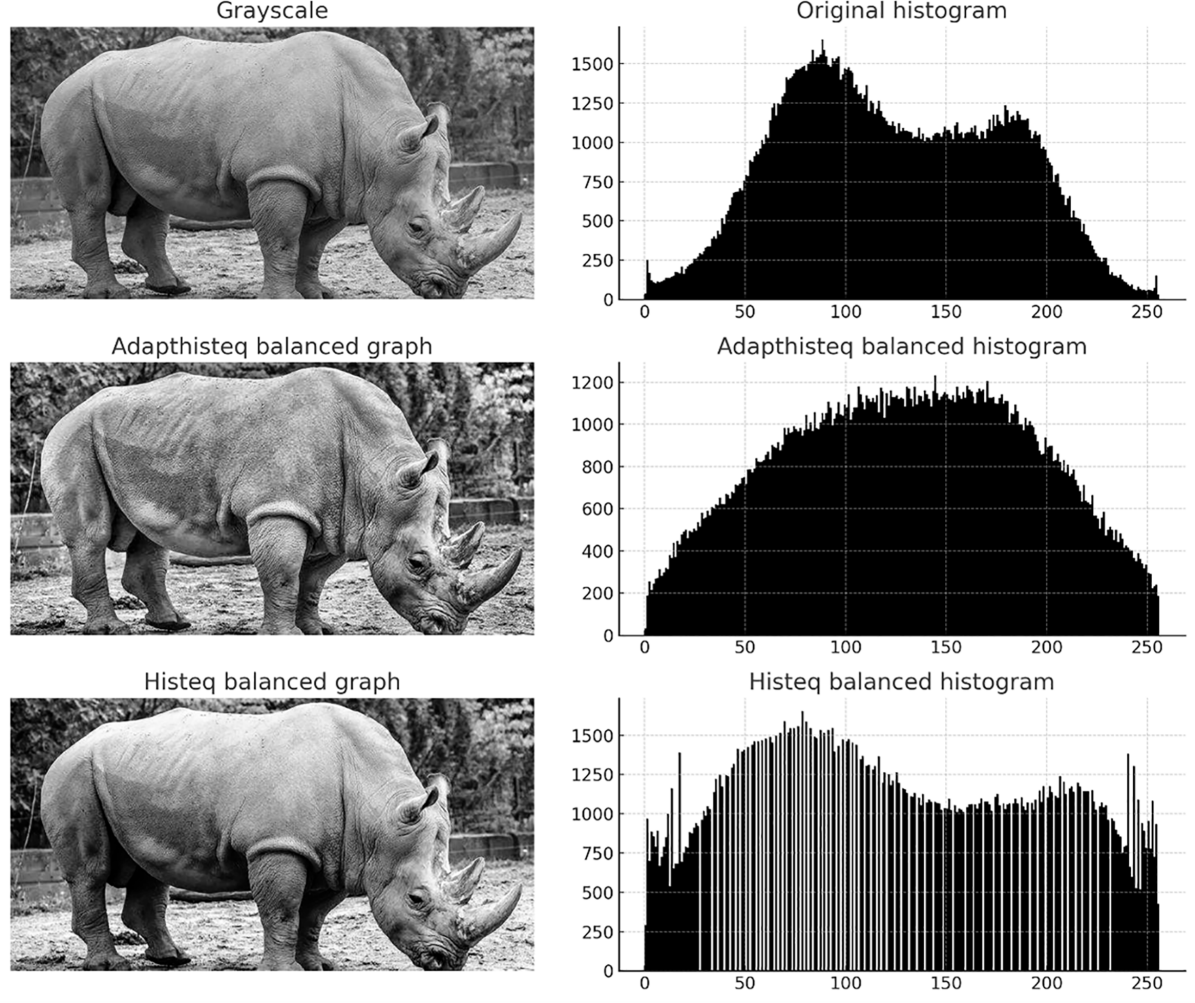
Histogram equalization process of rhinoceros images (adaptive histogram equalization and global histogram equalization). Republished from images.cv, Rhinoceros Image Classification Dataset under a CC BY 4.0 license, with permission from images.cv, original copyright 2020.

Common histogram equalization methods include adaptive histogram equalization and global histogram equalization [25]. Fig 2 shows the process of histogram equalization of museum collection images.

In Fig 2, two histogram equalization methods are described. Adaptive histogram equalization can better transform the original histogram into a uniformly distributed histogram, and its results can effectively improve image clarity. Therefore, adaptive histogram equalization can process museum collection images to enhance image contrast.

Based on the extracted features of the collection image and the results of fuzzy set processing, the similarity between the original image and the enhanced image of the collection can be analyzed. Through super-resolution reconstruction technology, the image can be explicitly reconstructed to enhance the details and visual effects of the museum collection images.

Museum collection image enhancement technology based on fuzzy sets can effectively describe the uncertainty and ambiguity issues in the image enhancement process. The degree of similarity of collection images before and after processing can be analyzed by converting the grayscale level into a fuzzy set. The dissimilar pixels are targeted for inpainting after determining the similar pixels of the collection image. Finally, the fuzzy set of the enhanced collection images is converted back into the grayscale values of the images, resulting in the final enhanced image.

**Fig 3:**
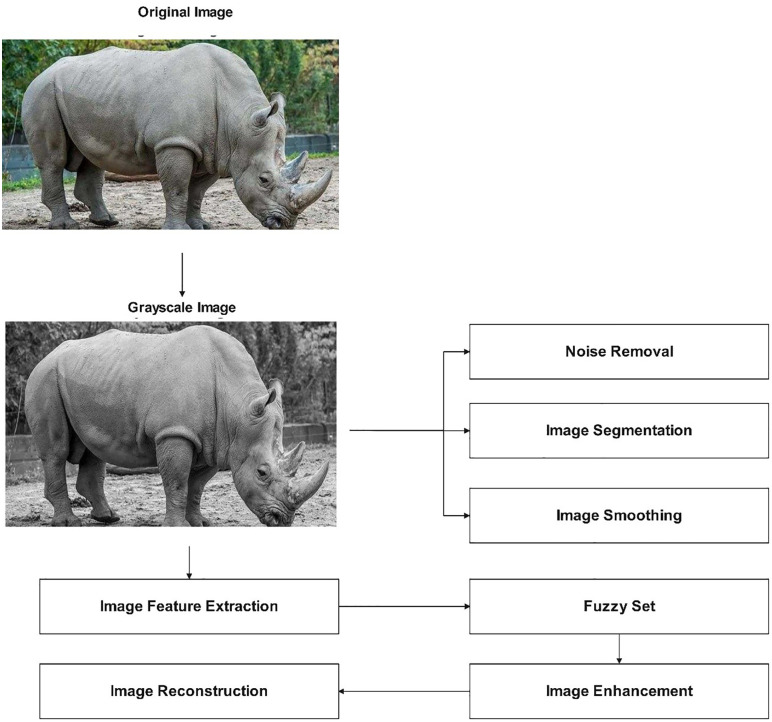
Enhance museum collection images using fuzzy set theory. Remove noise, extract features, apply fuzzy set analysis, and modify pixels for sharper, more defined results.

The image enhancement process of museum collections based on a fuzzy set is shown in Fig 3.

There are various steps in the fuzzy set image-enhancing process for museum collections, as shown in Fig 3. The photos are first preprocessed to get rid of extraneous info and noise. Doing this step ensures that the remaining data is correct and pertinent to the picture. The preprocessed photos are then used to extract features. These elements might be the image’s defining color, texture, form, or other components. The picture data is subjected to a fuzzy set after feature extraction. A mathematical notion known as a fuzzy set makes working with imprecise or uncertain data possible. Here, the similarity between the image’s pixels is determined using the fuzzy set. Image improvement is achieved by identifying and using dissimilar pixels. Lastly, the altered pixel values are used to recreate the improved pictures. The final photographs are sharper and better defined, perfect for various applications like documentation, preservation, and art restoration. This study does not involve any ethical issues, and all authors have provided informed consent for participation and publication.

## Experiments and cases

Museum collection images are an important way to preserve and display cultural relics. Obtaining museum collection images through photography and preserving cultural relics in the form of images is, to a certain extent, the inheritance of cultural heritage. Cultural research on images can avoid direct contact with cultural relics and extend their lifespan.

Although there are many advantages in storing museum collections in the form of images, the image quality of museum collections is not very high due to factors such as their age and shooting lighting, which seriously affects people’s ability to understand the collection details clearly through images. To effectively improve the viewing effect of the collection and conduct more detailed research on the collection, it is necessary to enhance the image of museum collections.

The dataset used in this study comprises labeled rhinoceros images sourced from the publicly available Images.CV – Rhinoceros Labeled Image Dataset. This dataset includes high-quality images annotated for classification and object detection tasks, making it well-suited for evaluating image enhancement methods. The diverse image conditions within the dataset, such as variations in lighting, background, and pose, provide a robust foundation for testing the effectiveness of our fuzzy set-guided enhancement approach [26]. To effectively analyze the effect of image enhancement, the museum collection image enhancement technology based on a fuzzy set is compared with histogram equalization and wavelet transform image enhancement technology.

The details of the collected images of museum collections are shown in Table 2.

**Table 2:** Details of museum collection images.

Serial number	Collection type	Quantity	Percentage
1	Cultural relic	33	33%
2	Natural specimens	18	18%
3	Physical information	16	16%
4	Non physical record data	14	14%
5	Intangible cultural heritage	19	19%

Table 2 describes the details of the museum collection images collected. The highest proportion of Chinese object images collected is 33%, while the lowest proportion of nonphysical record data images is 14%.

The 100 museum collection images are divided into five groups for image enhancement testing, and each collection image is processed using three image enhancement techniques. Establishing appropriate evaluation indicators is necessary to effectively evaluate the enhancement effect of collectable images.

The structural similarity index can be used to evaluate the fidelity of original and enhanced images. It comprehensively evaluates brightness similarity, contrast similarity, and structural similarity and evaluates the fidelity of images before and after enhancement through quantitative calculation and analysis [27].

Using structural similarity indicators for evaluation can effectively avoid the differences caused by subjective evaluation, where brightness similarity reflects the consistency of brightness information before and after image enhancement in the collection. The calculation formula for brightness similarity is:


$$L(x,y)=\frac{\text{2}\mu_{x}\mu_{y}+C_{\text{1}}}{\mu_{x}^{\text{2}}+\mu_{y}^{\text{2}}+C_{\text{1}}}$$ (8)

In Formula (8), *x* and *y* represent the original images and the enhanced images, respectively, while $\mu_{x}$ and $\mu_{y}$ represent the mean of *x* and *y*.

The formula for calculating contrast similarity is:


$$C(x,y)=\frac{\text{2}r_{x}r_{y}+C_{\text{2}}}{r_{x}^{\text{2}}+r_{y}^{\text{2}}+C_{\text{2}}}$$ (9)

In Formula (9), $r_{x}$ and $r_{y}$ represent the standard deviations of *x* and *y*, respectively.

The calculation formula for structural similarity is:


$$S(x,y)=\frac{r_{xy}+C_{\text{3}}}{r_{x}r_{y}+C_{\text{3}}}$$ (10)

In Formula (10), $r_{xy}$represents the covariance of *x* and *y*.

Then, the structural similarity index can be expressed as:


SSIM(x,y)=L(x,y)×C(x,y)×S(x,y) (11)

The structural similarity index’s value range is generally between 0 and 1. The larger the value, the higher the similarity of the collection images before and after enhancement processing, which means the higher the fidelity of the images after enhancement processing.

The purpose of image enhancement processing on museum collection images is to make the collection images clearer. The contrast of the images can reflect the clarity of the target and background in the enhanced images. The calculation of contrast is:


$$CR=\frac{{MAX}_{L}-{MIN}_{L}}{{MAX}_{L}+{MIN}_{L}}$$ (12)

In Formula (12), *CR* represents the contrast of museum collection images after enhancement.

The special values of image contrast are shown in Table 3.

**Table 3:** Meaning of special values for image contrast.

Serial number	Contrast ratio	Difference between light and dark areas
1	0	Very small
2	0.25	Less
3	0.5	Moderate
4	0.75	Relatively large
5	1	Very large

Table 3 describes the special values of image contrast. The range of image contrast values is between [0,1]. The higher the contrast, the more significant the difference between the light and dark areas in the image and the more precise the image.

## Results of image enhancement for museum collections

### Results of structural similarity indicators

The quality of museum collection images obtained through photography is not very high. Fuzzy set-based image enhancement technology can improve image quality, allowing museum collections to be better displayed, protected, and studied. The structural similarity index can comprehensively analyze the fidelity of the original collection images and the enhanced images.

In this paper’s museum collection image enhancement method based on fuzzy set theory, the choice of loss function is crucial to optimize image quality and improve the enhancement effect. This paper adopts a joint optimization strategy of multiple loss functions to ensure that the enhanced image maintains high fidelity in brightness, contrast, and structural details. Structural similarity loss is used to measure the similarity between the original image and the improved image to ensure that the key structural information of the image is not destroyed during the enhancement process. SSIM enables the improved image to maintain the overall visual characteristics of the original image through a comprehensive analysis of brightness, contrast, and structural features. Secondly, mean square error loss is used to calculate the pixel-level error to minimize the numerical deviation between the original image and the enhanced image, thereby improving the image accuracy. Contrast loss is introduced to enhance the contrast between bright and dark areas, improve the image’s visual quality, and make the details more straightforward. This paper adopts a weighted summation method to combine SSIM, MSE, and contrast loss to optimize the image enhancement effect comprehensively. This multi-loss fusion strategy not only improves the clarity and contrast of the image but also effectively retains the structural information of the original image so that the museum collection image can better meet the needs of cultural heritage protection and display after enhancement.

#### Luminance similarity.

**Fig 4:**
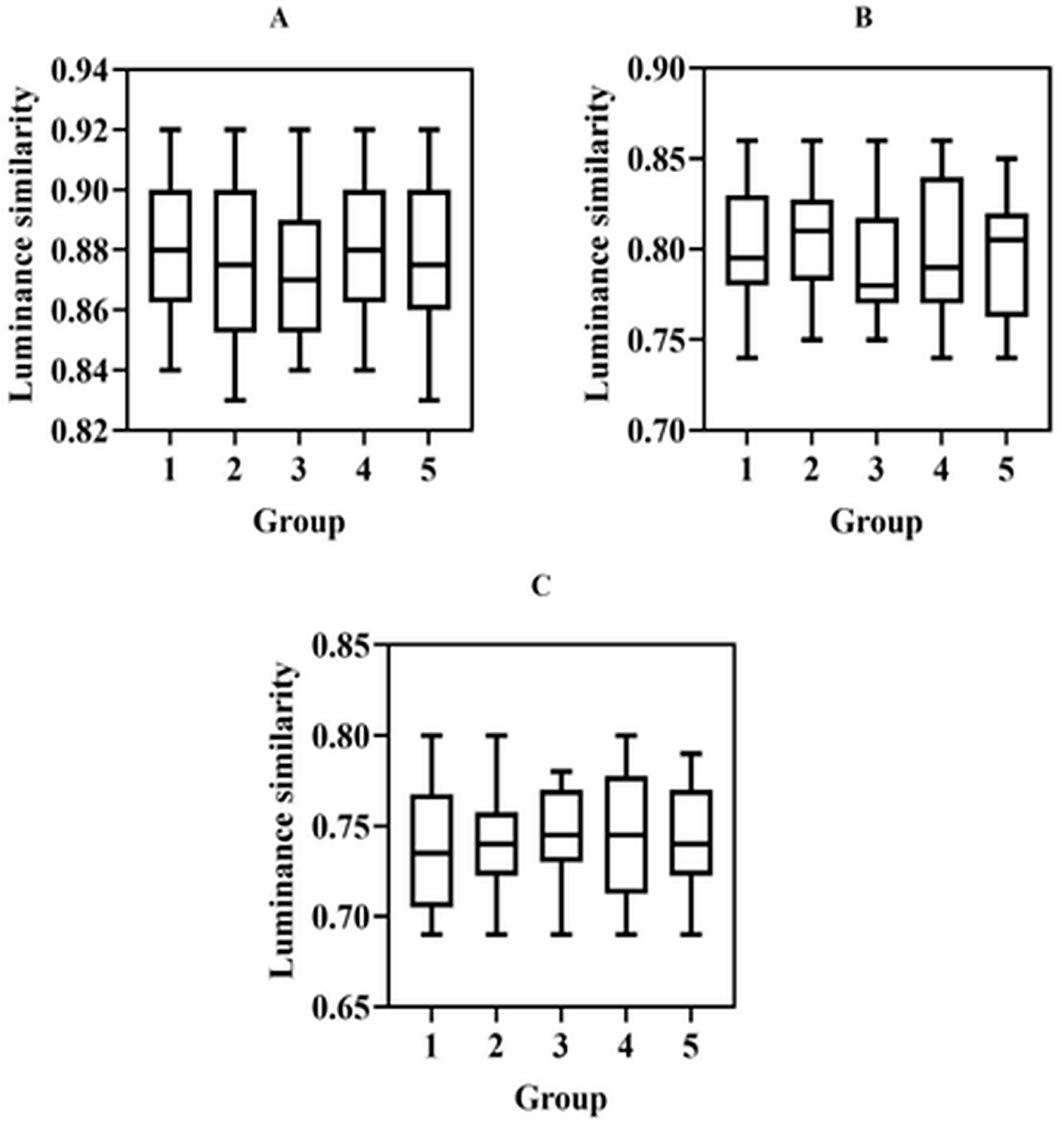
This figure compares the brightness similarity between the original image and the enhanced images obtained using three different techniques: fuzzy set image enhancement (A), histogram equalization (B), and wavelet transform (C).

The brightness similarity of images reflects the brightness similarity before and after image enhancement processing. Fig 4 compares the brightness similarity of museum collection images after image enhancement processing based on fuzzy set, histogram equalization, and wavelet transform.

Fig 4 describes the brightness similarity of the collection images before and after enhancement under three image enhancement techniques. The horizontal axis divides the tested museum collection images into 5 groups, while the vertical axis represents brightness similarity. The horizontal line in the box represents the median of the data. Comparing the data of (A), (B), and (C) in Fig 4, it was evident that the average brightness similarity in Fig 4(A) was greater than that in Fig 4(B), and the average brightness similarity in Fig 4(B) was greater than that in Fig 4(C). In Fig 4(A), the average brightness similarity was 0.88. Fig 4(B) had an average brightness similarity of 0.80, and Fig 4(C) had an average brightness similarity of 0.74.

Under the processing of three image enhancement techniques, the brightness similarity of museum collection images is relatively high. Image enhancement based on fuzzy sets can significantly improve brightness similarity, mainly because fuzzy sets can describe and process images’ uncertainty and fuzziness. The histogram equalization method can keep the histogram of the image evenly distributed at the gray level, but it is difficult to process the local details of the image. The museum collection image enhancement technology based on fuzzy sets can effectively improve the brightness similarity between the original images and the enhanced images of the collection.

#### Contrast similarity.

**Fig 5:**
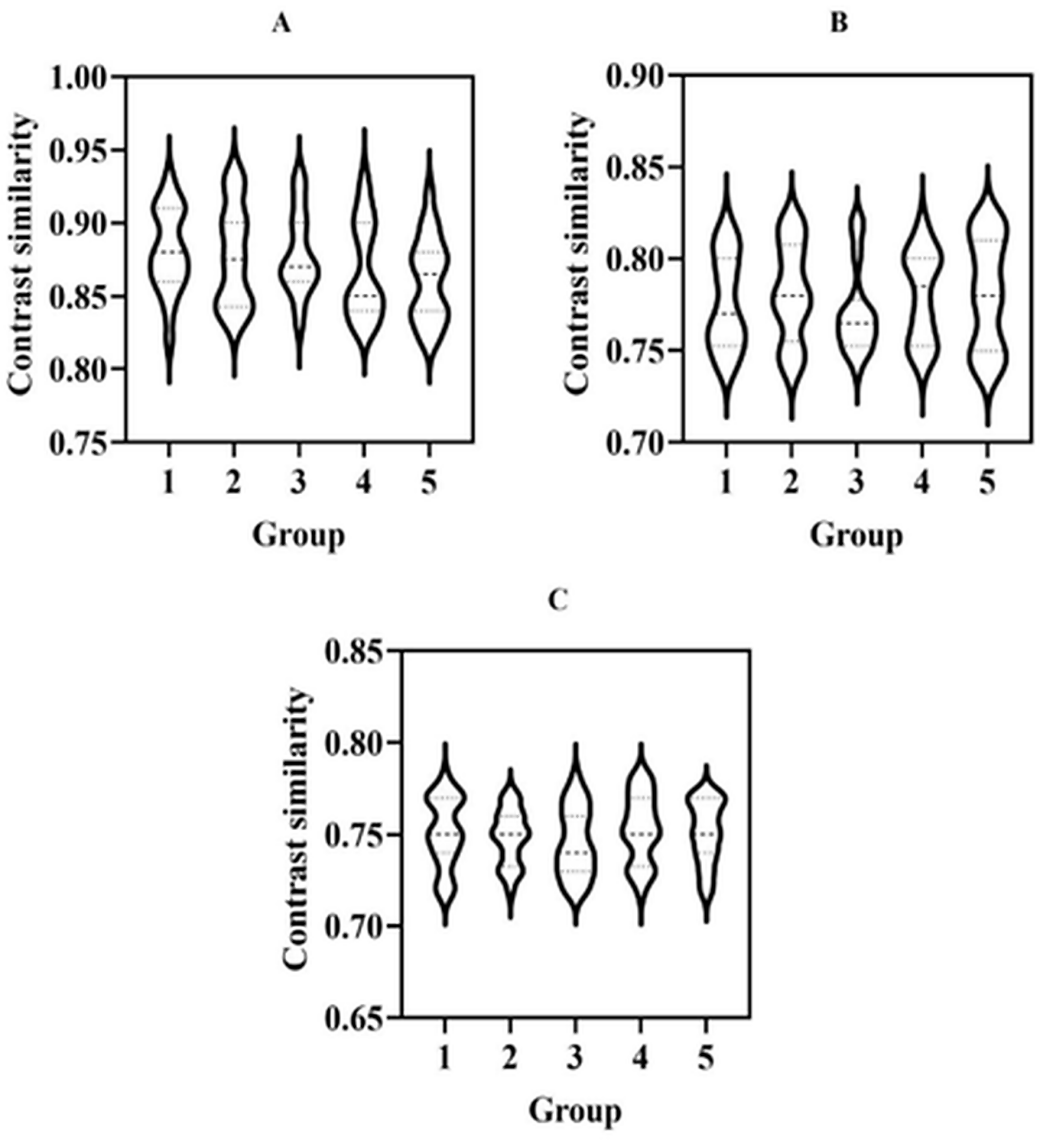
This figure compares the contrast similarity between the original image and the enhanced images obtained using three different techniques: (A) fuzzy set image enhancement, (B) histogram equalization, (C) and wavelet transform.

Contrast similarity measures the contrast similarity between an enhanced image and the original image by comparing the standard deviation of contrast [28]. Fig 5 shows the contrast similarity of museum collection images before and after processing using three image enhancement methods.

Fig 5 describes the contrast similarity of the collection images before and after enhancement under three image enhancement techniques. The horizontal axis represents the similarity of contrast between museum collection images before and after image enhancement processing, which are divided into 5 groups for testing. The average contrast similarity in Fig 5(A) was greater than that in Fig 5(B), and the average contrast similarity in Fig 5(B) was greater than that in Fig 5(C). The average contrast similarity in Fig 5(A), 5(B), and 5(C) was 0.87, 0.78, and 0.75, respectively.

Due to the influence of light and other factors, museum collections’ images are often unclear. The fuzzy set similarity algorithm can be used to analyze the similarity between the original image and the enhanced image through the fuzzy set-based image enhancement technology, and the contrast similarity before and after image enhancement can be significantly improved through targeted inpainting. However, histogram equalization and wavelet transformation make it challenging to describe the uncertainty in the image enhancement process.

#### Structural similarity.

**Fig 6:**
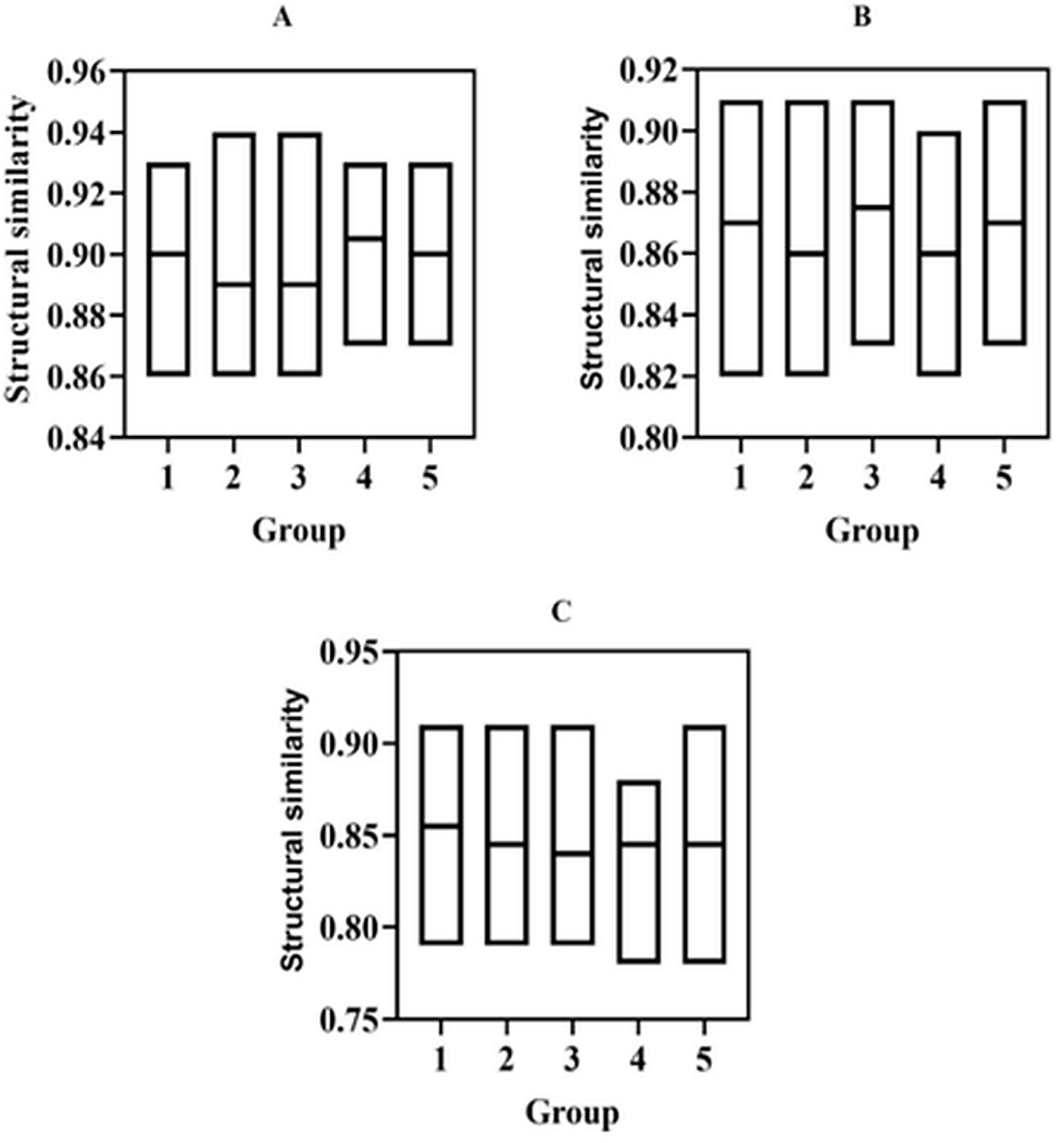
Compares the structural similarity between the original image and the enhanced images obtained using three different techniques: (A) fuzzy set image enhancement, (B) histogram equalization, and (C) wavelet transform.

Structural similarity is also a commonly used indicator for image quality evaluation. It can be used to analyze the degree of structural similarity of original museum collection images before and after enhancement processing. Fig 6 shows the structural similarity comparison results of museum collection images before and after processing using three image enhancement methods.

Fig 6 describes the structural similarity of the collection images before and after enhancement under three image enhancement techniques. The 100 collected images of museum collections were divided into five groups. The upper and lower boundary representation of the floating bar represent the maximum and minimum values of structural similarity in each group, and the middle horizontal line of the floating bar represents the median of the data. The average structural similarity in Fig 6(A) was greater than that in Fig 6(B), and the average structural similarity in Fig 6(B) was greater than that in Fig 6(C). The average structural similarity in Fig 6(A), 6(B), and 6(C) was 0.90, 0.87, and 0.85, respectively.

The grayscale level of the images of museum collections is transformed into a fuzzy set. Through the similarity algorithm, similar and dissimilar pixels are analyzed before and after image processing, and targeted image enhancement can effectively preserve the image structure in the original image. However, histogram equalization and wavelet transform image enhancement methods make it challenging to deal with the fuzziness of collection images. The similarity of brightness, contrast, and structure before and after the fuzzy set-based image enhancement technology processes museum collection images are higher than those of histogram equalization and wavelet transform algorithms, so the fuzzy set-based museum collection image enhancement has excellent fidelity [29,30].

### Results of contrast

**Fig 7:**
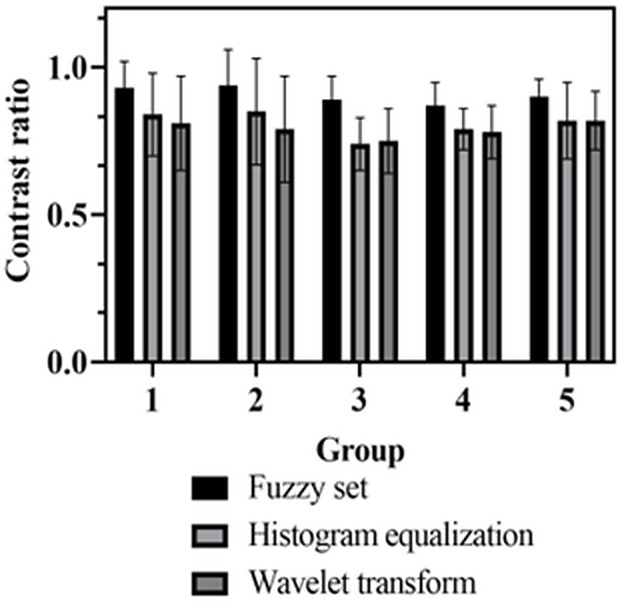
Contrast analysis results.

Contrast can evaluate the contrast between the target and background in the enhanced images. The higher the contrast of the enhanced images, the higher the clarity of the images. Fig 7 shows the contrast analysis results of museum collection images before and after processing using three image enhancement methods.

In Fig 7, the contrast of the enhanced collection images is described. The horizontal axis divides the collection images into five experimental groups, while the vertical axis represents the average contrast value. The contrast of museum collection image enhancement based on the fuzzy set was higher than that of the other two image enhancement methods in all five experiments. The average contrast of museum collections under the three enhancement modes of image enhancement based on a fuzzy set, histogram equalization, and wavelet transform were 0.91, 0.81, and 0.79, respectively.

The museum collection image enhancement based on a fuzzy set can maximize the image’s contrast, and the contrast of the two enhancement methods, histogram equalization and wavelet transform, is not much different. The museum collection image enhancement method based on fuzzy sets can effectively analyze the texture features in the collection images using fuzzy logic, thereby improving the clarity of the enhanced collection images.

### Image quality assessment

LPIPS is a perceptual quality assessment method based on deep learning. It uses a pre-trained deep network to extract image features and calculates the distance between the enhanced and original images in the feature space. NIQE is a reference-free image quality assessment indicator that evaluates the quality of an image based on a natural image statistical model without the need for an original reference image. This method calculates the naturalness score of an image by analyzing features such as local contrast and edge information of the image. FSIM measures the similarity between enhanced and original images by calculating the phase consistency and gradient amplitude. Unlike SSIM, which focuses mainly on brightness and contrast, FSIM focuses more on the image’s structural information and visual features. The comparison results are shown in Table 4.

**Table 4:** Image quality assessment.

Methods	LPIPS	NIQE	FSIM
Fuzzy set image enhancement	0.124	3.85	0.923
Histogram equalization	0.215	4.62	0.879
Wavelet transform	0.198	4.37	0.892
References [13]	0.175	4.12	0.901

From the evaluation results in Table 4, the fuzzy set image enhancement method performs best in all evaluation indicators. In terms of LPIPS, this method achieved the lowest value of 0.124, indicating that the enhanced image is closest to the original image in terms of perceptual quality, while the LPIPS value of the histogram equalization method is as high as 0.215, showing a large visual deviation. In terms of NIQE, the fuzzy set method’s 3.85 is lower than all the comparison methods, indicating that its enhanced image is closer to the natural, high-quality image, while the histogram equalization method has the highest NIQE value of 4.62, indicating that its enhanced image may be over-processed or artefacts. Regarding FSIM, the fuzzy set method has the highest value of 0.923, indicating that this method can better preserve the original image’s structural information and visual features. In summary, the fuzzy set image enhancement method is superior to traditional methods regarding visual fidelity, naturalness, and structural similarity, showing a more advanced enhancement effect.

## Conclusions

There are many uncertain factors in museum collection images. The gray value of pixels in museum collection images was converted into the corresponding fuzzy set. The collection images were analyzed and processed using a cosine similarity algorithm, and the dissimilar pixels were targeted for image enhancement. The effects of three different image enhancement techniques were compared. Image enhancement based on fuzzy sets can improve museum collections’ fidelity and clarity. However, this paper did not process large-scale museum collection images or consider the practicality of museum collection image enhancement technology based on fuzzy sets. In the future, this method will provide more possibilities and opportunities for the digital preservation and inheritance of information about cultural relics in museums.
